# Combined forced oscillation and fractional-order modeling in patients with work-related asthma: a case–control study analyzing respiratory biomechanics and diagnostic accuracy

**DOI:** 10.1186/s12938-020-00836-6

**Published:** 2020-12-09

**Authors:** Fábio Augusto d´Alegria Tuza, Paula Morisco de Sá, Hermano A. Castro, Agnaldo José Lopes, Pedro Lopes de Melo

**Affiliations:** 1 Biomedical Instrumentation Laboratory, Institute of Biology and Faculty of Engineering, State University of Rio de Janeiro, Haroldo Lisboa da Cunha Pavilion Number 104 and 105, São Francisco Xavier Street 524 Maracanã, Rio de Janeiro, RJ 20550-013 Brazil; 2 BioVasc Research Laboratory, Institute of Biology, State University of Rio de Janeiro, Rio de Janeiro, Brazil; 3 National School of Public Health, Oswaldo Cruz Foundation, Rio de Janeiro, Brazil; 4 School of Medical Sciences, Pulmonary Function Testing Laboratory, State University of Rio de Janeiro, Rio de Janeiro, RJ Brazil; 5 Rehabilitation Sciences Post-Graduation Program, Augusto Motta University Centre, Rio de Janeiro, Brazil

**Keywords:** Respiratory system modeling, Respiratory biomechanics, Respiratory impedance, Biomedical instrumentation, Extended RIC respiratory model, Fractional-order model, Forced oscillation technique, Respiratory oscillometry, Diagnostic of respiratory diseases, Bronchodilator, Work-related asthma

## Abstract

**Background:**

Fractional-order (FrOr) models have a high potential to improve pulmonary science. These models could be useful for biomechanical studies and diagnostic purposes, offering accurate models with an improved ability to describe nature. This paper evaluates the performance of the Forced Oscillation (FO) associated with integer (InOr) and FrOr models in the analysis of respiratory alterations in work-related asthma (WRA).

**Methods:**

Sixty-two individuals were evaluated: 31 healthy and 31 with WRA with mild obstruction. Patients were analyzed pre- and post-bronchodilation. The diagnostic accuracy was evaluated using the area under the receiver operating characteristic curve (AUC). To evaluate how well do the studied models correspond to observed data, we analyzed the mean square root of the sum (MSEt) and the relative distance (*R*_d_) of the estimated model values to the measured resistance and reactance measured values.

**Results and discussion:**

Initially, the use of InOr and FrOr models increased our understanding of the WRA physiopathology, showing increased peripheral resistance, damping, and hysteresivity. The FrOr model (AUC = 0.970) outperformed standard FO (AUC = 0.929), as well as InOr modeling (AUC = 0.838) in the diagnosis of respiratory changes, achieving high accuracy. FrOr improved the curve fitting (MSEt = 0.156 ± 0.340; *R*_d_ = 3.026 ± 1.072) in comparison with the InOr model (MSEt = 0.367 ± 0.991; *R*_d_ = 3.363 ± 1.098). Finally, we demonstrated that bronchodilator use increased dynamic compliance, as well as reduced damping and peripheral resistance.

**Conclusions:**

Taken together, these results show clear evidence of the utility of FO associated with fractional-order modeling in patients with WRA, improving our knowledge of the biomechanical abnormalities and the diagnostic accuracy in this disease.

## Background

Asthma is an umbrella label for various conditions characterized by chronic airway and/or lung disease. This condition includes several different phenotypes and is likely to have several different underlying mechanisms [1]. It is a treatable chronic airway disease that affects all age groups and has a high prevalence, morbidity, and mortality worldwide [2, 3].

Previous studies showed that 10 to 15% of adult-onset asthma cases are directly caused by occupational factors, while another 10% result from worsening pre-existing asthma due to workplace conditions. Work-related asthma (WRA) is characterized by obstructive airways and hyperreactivity due to conditions in the workplace rather than stimuli from outside the workplace. It is divided into two categories: occupational asthma (OA), attributed to the particular causes and conditions of the work environment, and exacerbated occupational asthma (EOA), referred to as pre-existing or concurrent asthma that is aggravated by occupational exposures. OA is one of the most prevalent occupational respiratory diseases in industrialized countries [3]. Occupational and environmental pollution in the form of dust, fumes, vapors, and toxic gases are essential risk factors for this disease.

Asthma functional assessment through spirometry establishes the diagnosis, documents the severity of airflow obstruction, and monitors the course of the disease and changes resulting from treatment [1]. However, this technique requires great patient cooperation in performing respiratory maneuvers, which may limit its use in the elderly or individuals with cognitive impairment [4]. Also, forced maneuver subjects the bronchi to stress, which can alter the bronchial tone, as well as exhaustion by repetition [5]. Whole-body plethysmography allows the measurement of lung volumes, capacities, and resistances [6, 7]. However, this method demands a cooperation maneuver similar to spirometry.

The Forced Oscillation Technique (FOT), also known as Oscillometry, is a system identification method used to evaluate the respiratory system resistance and reactance [8]. In this method, a sinusoidal pressure variation is applied in the opening of the airway through a mouthpiece, overlapping spontaneous ventilation. Thus, it requires only passive cooperation and no forced expiratory maneuvers are required [9]. Due to several recent technical enhancements, FOT currently represents state of the art in lung function evaluation [10]. Several authors have argued that it has the potential to improve diagnosis and monitor the treatment of respiratory diseases and that further studies are needed in this area [11, 12]. In this context, the FOT and the associated traditional parameters have been used to simplify the routine evaluation and to improve our understanding of the pathophysiology of several respiratory diseases [8]. A recent consensus statement pointed out that the use of FOT in occupational diseases requires further research [8].

A further improvement in the respiratory system evaluation is obtained by the use of inverse modeling. These models are based on electrical components analogous to the respiratory system properties of resistance, compliance, and inertance [13, 14]. It allows us to gain additional insight into the anatomical or pathophysiological changes that occur in respiratory diseases by obtaining detailed mechanical information about the respiratory system [10, 15, 16].

Recently, models based upon fractional derivatives and integrals have gained considerable popularity in the analysis of biological systems because they are more appropriate to describe the dynamic response of living systems than models based upon classical or integer-order derivatives and integrals [17–19]. In the particular case of the respiratory system, the ability of fractional-order models to effectively describe fractional power laws, hysteresis, and system memory is of pivotal importance. Parameters from these models can describe alterations in the distribution of gas flow within the system (spatial inhomogeneities), which are associated with peripheral abnormalities in the diseased lung. These parameters are also able to describe parenchymal deformity and elastance as well as changes in lung structure. Thanks to these characteristics, the fractional-order models have been the focus of much investigation in the search for improving respiratory clinical science and practice [10, 15, 16, 20].

In recent years, various fractional-order models have been developed and introduced [15, 21–24] to increase our understanding of cystic fibrosis [24], asthma in children [23], asbestos-exposed workers [25], chronic obstructive pulmonary disease [21, 22, 26], and sickle cell anemia [27]. Thus, there is a growing body of literature that recognizes the importance of fractional-order models in the interpretation of respiratory system functioning. The parameters obtained from these models hold the promise of improving the diagnosis and treatment of respiratory diseases.

Currently, however, these models show several unclear points, which resulted in much-debated topics, including the association of the model parameters and lung pathology [21], as well as the use of these parameters in the diagnostic of lung abnormalities [22]. The exact clarification of these points demands studies in well-defined groups of patients with different respiratory abnormalities [22]. The use of these models in asthma is limited to a preliminary study, including all asthma phenotypes [28]. This study, however, was not specific since it included all phenotypes and did not investigate the bronchodilator effect.

In this paper, we introduce integer and fractional-order models in the evaluation of the respiratory changes in work-related asthma and, for the first time, apply it to evaluate the bronchodilator response in these patients. This paper is organized as follows: first, the use of the new perspective of the FrOr model to interpret the changes in respiratory mechanics due to WRA will be evaluated. Then, we turn to the association between this model and the bronchodilator response in these patients. Finally, we evaluate the diagnostic accuracy of FrOr parameters and the best-performing parameters in the identification of the abnormal respiratory effects in WRA, as well as in the evaluation of the bronchodilator response in these patients.

## Results

Thirty-one asthmatics and 31 healthy volunteers were selected for this study. Their anthropometric data are presented in Table 1. Among the 31 individuals with WRA, 25 never smoked, 3 were former smokers (average smoking load = 3.2 pack-years), and 3 were smokers (average smoking load = 8.3 pack-years).

**Table 1:** Biometric characteristics (mean ± SD) of control individuals and patients with work-related asthma

	Control (*n* = 31)	Work-related asthma (*n* = 31)	*p*
Age (years)	51.0 ± 12.8	51.1 ± 12.9	ns
Body mass (kg)	70.5 ± 11.0	78.6 ± 14.8	*p* < 0.05
Height (cm)	163.3 ± 7.2	164.2 ± 7.1	ns
BMI (kg/m^2^)	25.9 ± 4.1	28.5 ± 6.3	*p *< 0.05
Gender (M/F)	14/17	14/17	–

The spirometric and plethysmographic characteristics of the studied subjects are described in Table 2. Considering the severity of airway obstruction, according to spirometric parameters [29], 19 patients were classified as mild (61%), nine were moderate (29%), while 3 (10%) presented severe obstruction. The analysis of obstruction severity by the plethysmographic resistance [6] showed that 45% of the patients were classified as normal, 26% had mild and moderate obstruction, while 29% presented severe obstruction.

**Table 2:** Spirometric and plethysmographic characteristics of control individuals and patients with work-related asthma

	Control (*n* = 31)	Work-related asthma before BD (*n* = 31)	Work-related asthma post BD (*n* = 31)
Spirometry			
FEV_1_ (L)	2.8 ± 0.7	2.3 ± 0.6**	2.5 ± 0.7^‡‡^
FEV_1_ (%)	93.1 ± 15.0	75.6 ± 19.2***	81.3 ± 17.4^‡‡‡‡^
FVC (L)	3.5 ± 0.8	3.1 ± 0.7	3.3 ± 0.8
FVC (%)	95.3 ± 15.1	84.1 ± 15.0**	86.6 ± 14.0^‡‡^
FEV_1_/FVC	79.9 ± 6.7	72.6 ± 11.7*	76.4 ± 10.2^‡^
FEF 25–75%	2.9 ± 1.1	2.0 ± 1.1**	2.4 ± 1.2
FEF/CVF	0.7 ± 0.2	0.6 ± 0.3	0.7 ± 0.3
EPF	7.1 ± 1.8	5.5 ± 2.0**	5.7 ± 2.2
Plethysmography			
VC (L)	3.73 ± 0.6	2.95 ± 0.7***	3.16 ± 0.6
IC (L)	2.45 ± 0.6	2.69 ± 0.6*	2.93 ± 0.6^‡^
ERV (L)	1.31 ± 0.08	0.33 ± 0.2***	0.26 ± 0.2
TLC (L)	5.48 ± 0.9	5.27 ± 1.1	5.14 ± 1.3
TV (L)	–	0.86 ± 0.3	0.87 ± 0.2
RV (L)	1.7 ± 0.3	2.3 ± 0.8***	2.0 ± 0.9^‡^
RV/TLC	31.11 ± 3.1	43.8 ± 12.9***	39 ± 11.6^‡^
FRC	3.02 ± 0.35	2.86 ± 1.2	2.3 ± 1.0^‡^
TGV	–	3.2 ± 1.3	2.7 ± 1.26^‡^
*R*_aw_	1.38 ± 0.09	5.8 ± 5.2***	3.7 ± 3.1^‡‡^
SGaw	0.24 ± 0.02	0.12 ± 0.09***	0.16 ± 0.1^‡^

Data are mean ± SD; % = percentage of the predicted values

FEV1: forced expiratory volume in the first second, FVC: forced vital capacity, FEF: forced expiratory flow between 25 and 75% of the FVC, %: percentage of the predicted values, EPF: expiratory peak flow, TLC: total lung capacity, FRC: functional residual capacity, ERV: expiratory reserve volume, RV: residual volume, *R*_aw_: airway resistance, TGV: thoracic gas volume, SGaw: specific airway resistance

*p < 0.05 related to control group

**p < 0.01 related to control group

***p < 0.001 related to control group

^‡^*p* < 0.05 related to pre-BD

^‡‡^*p* < 0.01 related to pre-BD

Figure 3 describes the results of the mean respiratory resistance (a) and reactance (b) curves in controls and patients with WRA pre- and post-bronchodilator (BD) use. WRA increased resistance values (Fig. 3a) and introduced more negative reactance values (Fig. 3b). The BD use resulted in a reduction in resistance (Fig. 3a) and less negative reactance values (Fig. 3b).

Table 3 shows the effects of WRA and BD use on the resistive and reactive properties of the respiratory system. A detailed graphical description of these results is provided in Additional file 1: Figure S1 and Additional file 2: Figure S2. Considering the resistive parameters, *R*_0_ and *R*_4_ increased significantly in WRA (*p* < 0.05), while *S* and *R*_20_ − *R*_4_ also presented significant changes (*p* < 0.0001). All of the reactive parameters showed significant changes in the comparison between the control group and the WRA pre-BD. Interestingly, the use of BD in WRA introduced significant changes (*p* < 0.01) in all of the studied reactive parameters.

**Table 3:** Oscillometric characteristics of control individuals and patients with work-related asthma and the effect of bronchodilator use

	Control (*n* = 31)	Work-related asthma before BD (*n* = 31)	Work-related asthma post BD (*n* = 31)
Resistive parameters			
*R*_0_ (cmH_2_O/L/s)	2.7 ± 0.7	3.9 ± 1.8*	2.8 ± 1.1^‡‡‡^
*R*_m_ (cmH_2_O/L/s)	2.6 ± 0.6	3.3 ± 1.3	2.5 ± 0.8^‡‡‡^
*S* (cmH_2_O/L/s^2^)	− 8.9 ± 18.7	− 66.0 ± 65****	− 32.7 ± 43.5^‡‡‡^
*R*_4_ (cmH_2_O/L/s)	2.6 ± 0.7	3.6 ± 1.5*	2.6 ± 0.9^‡‡‡^
*R*_12_ (cmH_2_O/L/s)	2.4 ± 0.6	3.0 ± 1.1	2.3 ± 0.8^‡‡‡^
*R*_20_ (cmH_2_O/L/s)	2.6 ± 0.7	2.8 ± 1.1	2.2 ± 0.8^‡‡‡^
*R*_4_− *R*_20_ (cmH_2_O/L/s)	0.03 ± 0.3	0.7 ± 0.9****	0.3 ± 0.5^‡‡^
Reactive parameters			
*X*_m_ (cmH_2_O/L/s)	0.4 ± 0.3	− 0.4 ± 0.9****	− 0.07 ± 0.6^‡‡‡^
Fr (Hz)	11.3 ± 1.5	20.0 ± 6.6****	17.2 ± 6.4^‡^
*C*_dyn_ (mL/cmH_2_O)	18.8 ± 6.3	14.4 ± 5.8**	19.4 ± 6.1^‡‡‡^
Axt (cmH_2_O/L)	8.7 ± 3.5	31.4 ± 30.1****	19.1 ± 22.0^‡‡‡^
Axi (cmH_2_O/L)	6.2 ± 2.6	20.4 ± 20.4****	11.9 ± 12.6^‡‡‡^
*Z*_4_ (cmH_2_O/L/s)	3.5 ± 0.9	5.0 ± 2.3*	3.6 ± 1.5^‡‡‡^

Data are mean ± SD

*R*_0_: intercept resistance; *R*_m_: mean resistance; *S*: slope of the resistive values; *R*_4_: resistance in 4 Hz; *R*_12_: resistance in 12 Hz; *R*_20_: resistance in 20 Hz; *X*_m_: mean reactance; Fr: resonance frequency; *C*_dyn_: dynamic compliance; Axt: area under the reactance curve using the approximation by a triangle; Axi: area under the reactance curve using the integral based on the trapezoidal rule; *Z*_4_: impedance modulus in 4 Hz

*p < 0.05 related to control group

**p < 0.01 related to control group

***p < 0.001 related to control group

****p < 0.0001 related to control group

^‡^*p* < 0.01 related to pre-BD

^‡‡^*p* < 0.001 related to pre-BD

^‡‡‡^*p* < 0.0001 related to pre-BD

Figure 4 displays the results obtained from the extended resistance–inertance–compliance (eRIC) model. The presence of abnormalities associated with WRA introduced significant increases in peripheral resistance (*R*_p_, *p* < 0.0001) and total resistance (*R*_t_, *p* < 0.01), as well as significant decreases in inertance (*I*, *p* < 0.001) and compliance (*C*, *p* < 0.01). In this figure, it is also apparent that the use of bronchodilator resulted in a significant decrease in all of the resistive parameters [central resistance (*R*), *p* < 0.0001; *R*_p_, *p* < 0.05; *R*_t_, *p* < 0.001] and a significant increase in *C* (*p* < 0.0001).

**Fig. 1:**
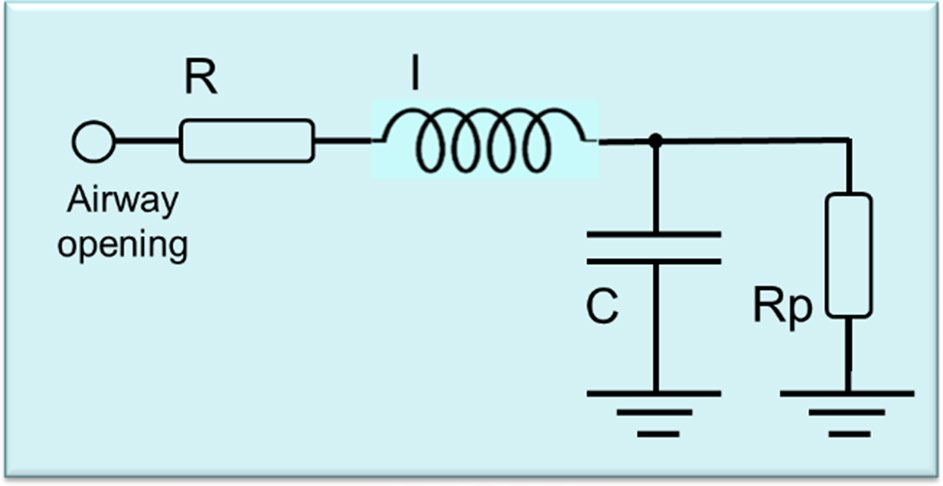
Extended RIC model used to interpret respiratory impedance. *R* reflects the central airway resistance, and *R*_p_ is related to peripheral resistance, while *I* and *C* are associated with respiratory inertance and compliance, respectively

The parameters obtained from the FrOr modeling are described in Fig. 5. As can be seen from this figure, *G* and *η* increased significantly in patients with WRA (Fig. 5a, *p* < 0.01 and Fig. 1c, *p* < 0.0001, respectively), while *H* showed a significant decrease (*p* < 0.0001, Fig. 5c). Besides, the use of bronchodilator resulted in a significant decrease in *G* (*p* < 0.05).

The errors analysis in the studied models is displayed in Table 4, which stands out the significant difference in *R*_d_ observed in patients before BD use.

**Table 4:** Mean square errors (MSE) and relative distances (*R*_d_) in the integer and fractional-order models studied in control individuals and patients with work-related asthma pre- and post-bronchodilator use

	MSEr (cmH_2_O/L/s)	MSEx (cmH_2_O/L/s)	MSEt (cmH_2_O/L/s)	*R*_d_ (%)
Control				
eRIC	0.073 ± 0.055	0.051 ± 0.036	0.094 ± 0.058	3.873 ± 1.465
FrOr	0.083 ± 0.131	0.055 ± 0.041	0.109 ± 0.130	3.900 ± 1.590
WRA pre				
eRIC	0.214 ± 0.892	0.237 ± 0.469	0.367 ± 0.991	3.363 ± 1.098
FrOr	0.102 ± 0.249	0.114 ± 0.234	0.156 ± 0.340	3.026 ± 1.072*
WRA post				
eRIC	0.039 ± 0.037	0.117 ± 0.281	0.132 ± 0.280	4.327 ± 1.616
FrOr	0.043 ± 0.049	0.088 ± 0.163	0.105 ± 0.166	5.509 ± 6.432

eRIC: extended resistance–inertance–compliance model; FrOr: fractional order; MSEr: real mean square error value; MSEx: imaginary mean square error value; MSEt: total mean square error value; *R*_d_: mean relative distance from the model and measured resistance and reactance; WRA: work-related asthma

* Indicates a significantly small error in the evaluated models

Table 5 shows that *R*_4_ − *R*_20_ presented the best correlations among spirometric and traditional FOT resistive parameters (*R* = − 0.66), while fr presented the highest correlation among the reactive parameters (*R* = − 0.66). Among the integer-order model parameters, *R*_p_ presented the highest association with spirometry (*R* = − 0.62), while among the FrOr model parameters, *η* showed the closest association (*R* = − 0.40). The degrees of these associations were reasonable to good.

**Table 5:** Correlation analysis among traditional, eRIC, and fractional-order parameters, and the spirometric results

	FEV_1_ (L)	FEV_1_ (%)	FVC (L)	FVC(%)	FEV_1_/FVC	FEF (L)	FEF25–75 (%)	FEF25–75/CVF
Resistive								
*R*_0_	− 0.33 ns	− 0.35 < 0.05	− 0.13 ns	− 0.11 ns	− 0.46 < 0.009	− 0.29 ns	− 0.44 < 0.02	− 0.43 < 0.02
*R*_m_	− 0.27 ns	− 0.27 ns	− 0.15 ns	− 0.12 ns	− 0.31 ns	− 0.30 ns	− 0.33 ns	− 0.29 ns
*S*	0.39 < 0.03	0.45 < 0.02	0.10 ns	0.12 ns	*0.65* < *0.0001*	0.22 ns	*0.58* *0.0005*	*0.62* *0.0001*
*R*_4_	− 0.30 ns	− 0.31 ns	− 0.12 ns	− 0.08 ns	− 0.43 < 0.02	− 0.28 ns	− 0.42 < 0.02	− 0.40 < 0.03
*R*_12_	− 0.29 ns	− 0.29 ns	− 0.16 ns	− 0.14 ns	− 0.32 ns	− 0.31 ns	− 0.35 < 0.05	− 0.30 ns
*R*_20_	− 0.17 ns	− 0.13 ns	− 0.16 ns	− 0.10 ns	− 0.07 ns	− 0.31 ns	− 0.05 ns	− 0.43 < 0.02
*R*_4_− *R*_20_	− 0.32 ns	− 0.37 < 0.04	− 0.01 ns	− 0.01 ns	− *0.66* < *0.0001*	− 0.09 ns	− *0.55* < *0.002*	− *0.64* < *0.0001*
Reactive								
*X*_m_	0.47 < 0.007	*0.53* < *0.003*	0.22 ns	0.24 ns	*0.65* < *0.0001*	0.24 ns	*0.60* *0.0002*	*0.62* *0.0001*
Fr	− *0.51* < *0.003*	− *0.56* *0.0008*	− 0.25 ns	− 0.32 ns	− *0.63* *0.0001*	− 0.34 ns	− *0.66* < *0.0001*	− *0.65* < *0.0001*
*C*_dyn_	0.31 ns	0.33 ns	0.25 ns	0.24 ns	0.24 ns	0.21 ns	0.35 ns	0.26 ns
Axt	− 0.43 < 0.02	− *0.49* < *0.005*	− 0.21 ns	− 0.24 ns	− *0.57* *0.0006*	− 0.23 ns	− *0.52* < *0.003*	− *0.52* < *0.003*
Axi	− 0.44 < 0.02	− *0.49* < *0.005*	− 0.20 ns	− 0.22 ns	− *0.61* *0.0002*	− 0.23 ns	− *0.54* < *0.002*	− *0.55* < *0.002*
*Z*_4_	− 0.36 < 0.05	− 0.39 < 0.03	− 0.18 ns	− 0.16 ns	− 0.47 < 0.008	− 0.25 ns	− 0.44 < 0.02	− 0.42 < 0.02
eRIC								
*R*	− 0.05 ns	0.008 ns	− 0.10 ns	− 0.02 ns	0.11 ns	− 0.26 ns	− 0.01 ns	0.08 ns
*R*_p_	− 0.37 < 0.04	− 0.41 < 0.02	− 0.10 ns	− 0.09 ns	− *0.62* *0.0001*	− 0.17 ns	− *0.53* < *0.002*	− *0.59* *0.0001*
*R*_t_	− 0.31 ns	− 0.31 ns	− 0.14 ns	− 0.09 ns	− 0.41 < 0.03	− 0.28 ns	− 0.42 < 0.02	− 0.40 < 0.03
*I*	0.37 < 0.05	0.42 < 0.02	0.14 ns	0.21 ns	*0.54* < *0.002*	0.14 ns	*0.48* < *0.006*	*0.53* < *0.002*
*C*	0.24 ns	0.30 ns	0.13 ns	0.17 ns	0.28 ns	0.14 ns	0.37 < 0.04	0.33 ns
FrOr								
*G*	− *0.38* < *0.0026*	− *0.35* *0.0058*	− 0.30 < 0.02	− 0.32 < 0.02	− 0.14 ns	− 0.33 < 0.009	− 0.28 < 0.026	− 0.12 ns
*H*	0.12 ns	0.07 ns	− 0.11 ns	0.00 ns	0.16 ns	013 ns	0.15 ns	0.15 ns
*η*	− *0.40* < *0.002*	− 0.28 < 0.03	− 0.32 < 0.02	− 0.19 ns	− *0.36* < *0.004*	− 0.31 < 0.02	− 0.34 < 0.007	− 0.33 < 0.009

Significance was analyzed after Bonferroni correction. The significant associations are described in italic

FEV1: forced expiratory volume in the first second; FVC: forced vital capacity; FEF: forced expiratory flow between 25 and 75% of the FVC; %: percentage of the predicted values; *R*_0_: intercept resistance; *R*_m_: mean resistance; *S*: slope of the resistive values; *R*_4_: resistance in 4 Hz; *R*_12_: resistance in 12 Hz; *R*_20_: resistance in 20 Hz; *X*_m_: mean reactance; Fr: resonance frequency; *C*_dyn_: dynamic compliance; Axt: area under the reactance curve using the approximation by a triangle; Axi: area under the reactance curve using the integral based on the trapezoidal rule; *Z*_4_: impedance modulus in 4 Hz; eRIC: extended resistance–inertance–compliance model; FrOr: fractional order; *R*: central airway resistance; *R*_p_: peripheral resistance; *R*_t_: total resistance; *I*: pulmonary inertance; *C*: alveolar compliance; *G*: damping factor; *H*: elastance; *η*: hysteresivity coefficient

The results of the correlation analysis among FOT, eRIC, and FrOr parameters and the plethysmographic measurements are described in Table 6. Reasonable to good associations were observed. *S* showed the closest correlations among plethysmographic and traditional FOT resistive parameters (*R* = − 0.57), while *X*_m_ presented the best correlation among the reactive parameters (*R* = − 0.58). Among the integer-order model parameters, *R*_p_ presented the best association with plethysmography (*R* = − 0.50), while among the FrOr model parameters, *η* and *G* showed the highest correlation (*R* = − 0.53).

**Table 6:** Association among traditional, eRIC and fractional-order parameters and plethysmographic analysis

	VC (L)	IC (L)	ERV (L)	TV (L)	RV/TLC	FRC (L)	RV (L)	TLC (L)	TGV (L)	*R*_aw_	SGaw
Resistive											
*R*_0_	− 0.36 < 0.05	− 0.41 < 0.03	− 0.09 ns	− 0.34 ns	0.05 ns	− 0.20 ns	0.03 ns	− 0.31 ns	− 0.20 ns	0.35 ns	− 0.30 ns
*R*_m_	− 0.39 < 0.03	− 0.41 < 0.02	− 0.14 ns	− 0.34 ns	0.02 ns	− 0.25 ns	− 0.20 ns	− 0.37 < 0.04	− 0.23 ns	0.21 ns	− 0.17 ns
*S*	0.22 ns	0.30 Ns	− 0.0005 ns	0.25 ns	− 0.09 ns	0.06 ns	− 0.004 ns	0.09 ns	0.09 ns	− *0.57* *0.0007*	*0.51* < *0.003*
*R*_4_	− 0.37 < 0.04	− 0.43 < 0.02	− 0.08 ns	− 0.34 ns	0.06 ns	− 0.19 ns	− 0.15 ns	− 0.32 ns	− 0.18 ns	0.28 ns	− 0.28 ns
*R*_12_	− 0.39 < 0.03	− 0.41 < 0.02	− 0.12 ns	− 0.32 ns	0.01 ns	− 0.25 ns	− 0.19 ns	− 0.37 ns	− 0.23 ns	0.21 ns	− 0.20 ns
*R*_20_	− 0.43 < 0.02	− 0.42 < 0.02	− 0.18 ns	− 0.34 ns	0.008 ns	− 0.28 ns	− 0.24 ns	− 0.44 < 0.02	− 0.24 ns	− 0.02 ns	0.03 ns
*R*_4_ − *R*_20_	− 0.11 ns	− 0.23 ns	0.08 ns	− 0.16 ns	0.1 ns	0.02 ns	0.03 ns	− 0.008 ns	− 0.01 ns	*0.52* < *0.003*	− *0.54* < *0.002*
Reactive											
*X*_m_	0.26 ns	0.29 ns	0.03 ns	0.22 ns	− 0.13 ns	0.006 ns	− 0.01 ns	0.09 ns	0.02 ns	− *0.58* *0.0005*	*0.49* < *0.006*
Fr	− 0.38 < 0.04	− 0.39 < 0.03	− 0.06 ns	− 0.30 ns	0.30 ns	0.18 ns	0.10 ns	− 0.04 ns	0.13 ns	0.40 < 0.03	− 0.38 < 0.04
*C*_dyn_	0.40 < 0.03	0.38 < 0.04	0.22 ns	0.30 ns	0.08 ns	0.37 < 0.05	0.20 ns	0.36 < 0.05	0.36 < 0.05	− 0.30 ns	0.24 ns
Axt	− 0.25 ns	− 0.28 ns	− 0.07 ns	− 0.24 ns	0.07 ns	− 0.09 ns	− 0.04 ns	− 0.15 ns	− 0.09 ns	*0.56* *0.0008*	− 0.41 < 0.03
Axi	− 0.22 ns	− 0.27 ns	− 0.05 ns	− 0.23 ns	0.05 ns	− 0.09 ns	− 0.04 ns	− 0.13 ns	− 0.09 ns	*0.59* *0.0004*	− 0.46 < 0.008
*Z*_4_	− 0.32 ns	− 0.36 < 0.05	− 0.11 ns	− 0.30 ns	0.02 ns	− 0.21 ns	− 0.13 ns	− 0.27 ns	− 0.20 ns	0.44 < 0.02	− 0.33 < 0.07
eRIC											
*R*	− 0.37 < 0.04	− 0.35 ns	− 0.20 ns	− 0.30 ns	− 0.05 ns	− 0.32 ns	− 0.30 ns	− 0.46 < 0.008	− 0.28 ns	− 0.20 ns	0.16 ns
*R*_p_	− 0.21 ns	− 0.33 ns	0.09 ns	− 0.23 ns	0.10 ns	− 0.01 ns	− 0.009 ns	− 0.09 ns	− 0.03 ns	*0.50* < *0.004*	− 0.48 < 0.007
*R*_t_	− 0.38 < 0.04	− 0.46 < 0.01	− 0.04 ns	− 0.35 < 0.05	0.04 ns	− 0.19 ns	− 0.18 ns	− 0.34 ns	− 0.18 ns	0.27 ns	− 0.27 ns
*I*	0.10 ns	0.09 ns	− 0.01 ns	0.06 ns	− 0.25 ns	− 0.27 ns	− 0.14 ns	− 0.13 ns	− 0.25 ns	− 0.41 < 0.02	0.35 < 0.05
*C*	0.30 ns	0.32 ns	0.19 < 0.04	0.21 ns	0.13 ns	0.39 < 0.03	0.20 ns	0.32 ns	0.45 < 0.02	− 0.41 < 0.03	0.37 < 0.04
FrOr											
*G*	− 0.15 ns	− 0.16 ns	− 0.16 ns	− 0.20 ns	− 0.15 ns	− 0.34 ns	− 0.22 ns	− 0.25 ns	− 0.32 ns	*0.53* < *0.003*	− 0.31 ns
*H*	− 0.31 ns	− 0.31 ns	− 0.14 ns	− 0.27 ns	0.07 ns	− 0.16 ns	− 0.07 ns	− 0.25 ns	− 0.13 ns	0.36 < 0.05	− 0.16 ns
*η*	0.13 ns	0.15 ns	− 0.16 ns	− 0.05 ns	− 0.19 ns	− 0.16 ns	− 0.08 ns	0.03 ns	− 0.24 ns	*0.53* < *0.003*	− 0.34 ns

Significance was analyzed after Bonferroni correction. The significant associations are described in italic

FEV1: forced expiratory volume in the first second; FVC: forced vital capacity; FEF: forced expiratory flow between 25 and 75% of the FVC; %: percentage of the predicted values; *R*_0_: intercept resistance; *R*_m_: mean resistance; *S*: slope of the resistive values; *R*_4_: resistance in 4 Hz; *R*_12_: resistance in 12 Hz; *R*_20_: resistance in 20 Hz; *X*_m_: mean reactance; Fr: resonance frequency; *C*_dyn_: dynamic compliance; Axt: area under the reactance curve using the approximation by a triangle; Axi: area under the reactance curve using the integral based on the trapezoidal rule; *Z*_4_: impedance modulus in 4 Hz; eRIC: extended resistance–inertance–compliance model; FrOr: fractional order; *R*: central airway resistance; *R*_p_: peripheral resistance; *R*_t_: total resistance; *I*: pulmonary inertance; *C*: alveolar compliance; *G*: damping factor; *H*: elastance; *η*: hysteresivity coefficient

Three of the studied traditional FOT parameters presented adequate diagnostic accuracy (0.8 < AUC < 0.9) to identify respiratory abnormalities in patients with WRA (Table 7: *S*, *X*_m_, Axt, and Axi). This initial ROC analysis also showed that fr was able to achieve high diagnostic accuracy (AUC > 0.9).

**Table 7:** Diagnostic accuracy (mean and 95% confidence interval) of the traditional FOT parameters in the diagnostic of respiratory abnormalities in patients with work-related asthma

	AUC	Se (%)	Sp (%)	Cut-off
*R*_0_	0.687 0.556–0.799	45.1 27.3–64.0	93.5 78.6–99.2	> 3.53
*R*_m_	0.634 0.502–0.753	35.4 19.2–54.6	96.7 83.3–99.9	> 3.54
*R*_4_	0.673 0.542–0.787	38.7 21.8–57.8	96.7 83.3–99.9	> 3.70
*R*_12_	0.635 0.503–0.753	38.7 21.8–57.8	87.1 70.2–96.4	> 3.13
*R*_20_	0.513 0.383–0.642	100 88.8–100.0	12.9 3.6–29.8	> 1.49
*S*	*0.840* 0.745–0.934	67.7 48.6–83.3	80.6 62.5–92.5	≤ − 23.3
*R*_4_ − *R*_20_	0.795 0.673–0.887	80.6 62.5–92.5	64.5 45.4–80.8	> 0.16
*X*_m_	*0.826* 0.724–0.929	67.7 48.6–83.3	87.1 70.2–96.4	≤ − 0.03
Fr	*0.929* 0.835–0.979	74.2 55.4–88.1	100 88.8–100.0	> 14.7
*C*_dyn_	0.695 0.565–0.806	48.3 30.2–66.9	90.3 74.2–98.0	≤ 12.41
Axt	*0.875* 0.767–0.945	70.9 52.0–85.8	96.7 83.3–99.9	> 13.56
Axi	*0.819* 0.701–0.906	80.6 62.5–92.5	67.7 48.6–83.3	> 7.08
*Z*_4_	0.690 0.558–0.822	48.3 30.2–66.9	87.1 70.2–96.4	> 4.54

Values considered adequate are described in italic

AUC: area under the receiver operating curve; Se: sensitivity; Sp: specificity; *R*_0_: intercept resistance; *R*_m_: mean resistance; *S*: slope of the resistive values; *R*_4_: resistance in 4 Hz; *R*_12_: resistance in 12 Hz; *R*_20_: resistance in 20 Hz; *X*_m_: mean reactance; Fr: resonance frequency; *C*_dyn_: dynamic compliance; Axt: area under the reactance curve using the approximation by a triangle; Axi: area under the reactance curve using the integral based on the trapezoidal rule; *Z*_4_: impedance modulus in 4 Hz. eRIC: extended resistance–inertance–compliance model; FrOr: fractional order; *R*: central airway resistance; *R*_p_: peripheral resistance; *R*_t_: total resistance; *I*: pulmonary inertance; *C*: alveolar compliance; *G*: damping factor; *H*: elastance; *η*: hysteresivity coefficient

Table 8 provides the results of the ROC analysis performed using the eRIC and FrOr models. *R*_p_ was the only parameter obtained using the eRIC model able to achieve adequate diagnostic accuracy. Considering the parameters obtained by the FrOr model, *H* achieved adequate diagnostic performance, while *η* showed high diagnostic accuracy.

**Table 8:** Diagnostic accuracy (mean and 95% confidence interval) of the eRIC and fractional-order parameters in the detection of respiratory alterations in patients with work-related asthma

	AUC	Se (%)	Sp (%)	Cut-off
eRIC				
*R*	0.514 0.384–0.643	74.2 55.4–88.1	41.9 24.5–60.9	≤ 2.87
*R*_t_	0.703 0.574–0.813	41.9 24.5–60.9	96.7 83.3–99.9	> 3.68
*R*_p_	*0.838* 0.734–0.942	67.7 48.6–83.3	96.8 83.3–99.9	> 0.80
*I*	0.746 0.619–0.848	64.5 45.4–80.8	80.6 62.5–92.5	≤ 0.007
*C*	0.747 0.621–0.849	58.0 39.1–75.5	93.5 78.6–99.2	≤ 0.01
FrOr				
*G*	0.729 0.602 to 0.856	74.19 55.4–88.1	58.06 39.1–75.5	> 13.53
*H*	*0.881* 0.785–0.978	83.87 66.3–94.5	90.33 74.2–98.0	< 23.12
*η*	*0.970* 0.915–1.000	96.77 83.3–99.9	96.77 83.3–99.9	> 0.658

Values considered adequate are described in italic

AUC: area under the receiver operator curve; Se: sensibility; Sp: specificity; eRIC: extended resistance–inertance–compliance model; FrOr: fractional order; *R*: central airway resistance; *R*_p_: peripheral resistance; *R*_t_: total resistance; *I*: pulmonary inertance; *C*: alveolar compliance; *G*: damping factor; *H*: elastance; *η*: hysteresivity coefficient

Figure 6a shows the performance of the best parameters obtained from traditional FOT analysis, eRIC, and FrOr modeling to identify respiratory changes in WRA. The AUCs of fr and *η* were significantly higher than *R*_p_ (*p* < 0.04 and *p* < 0.02, respectively), while the AUCs of fr and *η* were not significantly different. The results of the LOOCV analysis in the most discriminative parameters observed in the detection of respiratory abnormalities in WRA using traditional parameters (Table 7), the eRIC, and the FrOr model (Table 8), are described in Fig. 6b. Fr achieved adequate diagnostic value, while *η* obtained a high accuracy.

The ability of the traditional FOT parameters to detect the changes due to bronchodilator use in patients with WRA is described in Table 9. Similar analyses for the eRIC and FrOr models are presented in Table 10.

**Table 9:** Area under the receiving operator curve (mean and 95% confidence interval) of the traditional FOT parameters in the identification of the bronchodilator response in patients with work-related asthma

	AUC	Se (%)	Sp (%)	Cut-off
*R*_0_	0.699 0.570–0.829	74.1 55.4–88.1	58.0 39.1–75.5	> 2.72
*R*_m_	0.694 0.563–0.825	83.8 66.3–94.5	51.6 33.1–69.8	> 2.39
*R*_4_	*0.708* 0.580–0.835	93.5 78.6–99.2	38.7 21.8–57.8	> 2.00
*R*_12_	0.690 0.558–0.822	64.5 45.4–80.8	70.9 52.0–85.8	> 2.46
*R*_20_	0.691 0.558–0.824	74.1 55.4–88.1	61.2 42.2–78.2	> 2.20
*S*	0.686 0.554–0.817	77.4 58.9–90.4	51.61 33.1–69.8	≤ − 19.9
*R*_4_ − *R*_20_	0.677 0.542–0.813	61.2 42.2–78.2	70.9 52.0–85.8	> 0.30
*X*_m_	0.629 0.489–0.768	45.1 27.3–64.0	87.1 70.2–96.4	≤ − 0.35
Fr	0.634 0.494–0.773	51.6 33.1–69.8	77.4 58.9–90.4	> 19.5
*C*_dyn_	*0.706* 0.574–0.837	58.0 39.1–75.5	87.1 70.2–96.4	≤ 14.3
Axt	0.697 0.566–0.828	51.6 33.1–69.8	83.8 66.3–94.5	> 18.6
Axi	0.665 0.530–0.800	61.29 42.2 – 78.2	64.52 45.4 – 80.8	≤ 9.10
*Z*_4_	*0.726* 0.601–0.851	74.1 55.4–88.1	61.2 42.2–78.2	> 3.36

Values considered adequate are described in italic

AUC: area under the receiver operating curve; Se: sensitivity; Sp: specificity; *R*_0_: intercept resistance; *R*_m_: mean resistance; *S*: slope of the resistive values; *R*_4_: resistance in 4 Hz; *R*_12_: resistance in 12 Hz; *R*_20_: resistance in 20 Hz; *X*_m_: mean reactance; Fr: resonance frequency; *C*_dyn_: dynamic compliance; Axt: area under the reactance curve using the approximation by a triangle; Axi: area under the reactance curve using the integral based on the trapezoidal rule; *Z*_4_: impedance modulus in 4 Hz

**Table 10:** Diagnostic accuracy (mean and 95% confidence interval) of the eRIC and fractional-order parameters in the detection of respiratory effects of bronchodilator use in patients with work-related asthma

	AUC	Se (%)	Sp (%)	Cut-off
eRIC				
*R*	0.678 0.544–0.813	67.7 48.6–83.3	67.7 48.6–83.3	≤ 2.1
*R*_t_	0.664 0.529–0.799	93.6 78.6–99.2	32.2 16.7–51.4	> 2.59
*R*_p_	0.546 0.400–0.693	29.0 14.2–48.0	96.7 83.3–99.9	> 1.81
*I*	0.518 0.372–0.663	29.0 14.2–48.0	83.8 66.3–94.5	≤ 0.003
*C*	0.651 0.513–0.790	58.0 39.1–75.5	77.4 58.9–90.4	≤ 0.01
FrOr				
*G*	0.660 0.528–0.775	61.29 42.2–78.2	64.52 45.4–80.8	≤ 14.65
*H*	0.645 0.513–0.763	74.19 55.4–88.1	61.29 42.2–78.2	≤ 16.81
*η*	0.505 0.375–0.634	58.06 39.1–75.5	58.06 39.1–75.5	≤ 0.93

AUC: area under the receiver operator curve; Se: sensibility; Sp: specificity; eRIC: extended resistance–inertance–compliance model; FrOr: fractional order; *R*: central airway resistance; *R*_p_: peripheral resistance; *R*_t_: total resistance; *I*: pulmonary inertance; *C*: alveolar compliance; *G*: damping factor; *H*: elastance; *η*: hysteresivity coefficient

**Fig. 2:**
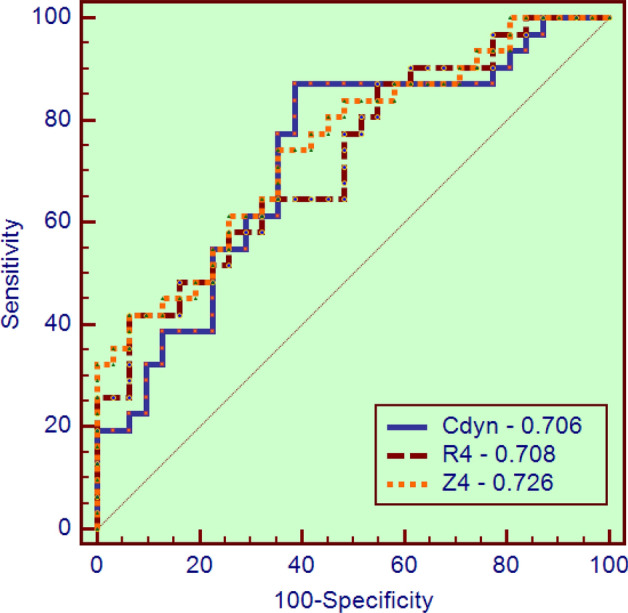
Receiver operator characteristic analysis and associated area under the curve in the three best parameters observed in the evaluation of the bronchodilator responses. *R*_4_: resistance in 4 Hz; *C*_dyn_: dynamic compliance and *Z*_4_: impedance modulus in 4 Hz

The results of the ROC analysis performed in the best parameters obtained from traditional FOT analysis (Table 9), and eRIC and FrOr modeling (Table 10) to identify bronchodilator responses in WRA are described in Fig. 2. LOOCV analysis considering these parameters was not able to reach adequate diagnostic values (AUC ≥ 0.7).

## Discussion

This is the first study that has quantitatively evaluated the performance of the eRIC model and a FrOr model in the analysis of airway obstruction and the bronchodilator response in work-related asthma. The most exciting findings of this study were as follows: (1) patients with WRA showed increased peripheral resistance, damping, and hysteresivity when compared with controls; (2) fractional-order analysis outperformed standard FOT, as well as integer-order modeling in the diagnosis of respiratory changes in these patients; (3) the bronchodilator use in WRA resulted in increased dynamic compliance and reduced damping and peripheral resistance; and (4) standard FOT analysis outperformed integer and fractional-order modeling in the identification of the bronchodilator effects in these patients.

Table 1 shows that the two studied groups were of comparable age, height, and gender distribution. Although there were slight differences between the groups related to body mass, this parameter is not determinants in terms of alterations in respiratory impedance. We should highlight the fact that the main parameter that has a significant impact on the impedance—subject height [30]—was quite similar between the groups under study. The changes in volumes and flows observed in WRA patients before and after BD use (Table 2) were consistent with the involved physiopathology [31].

**Fig. 3:**
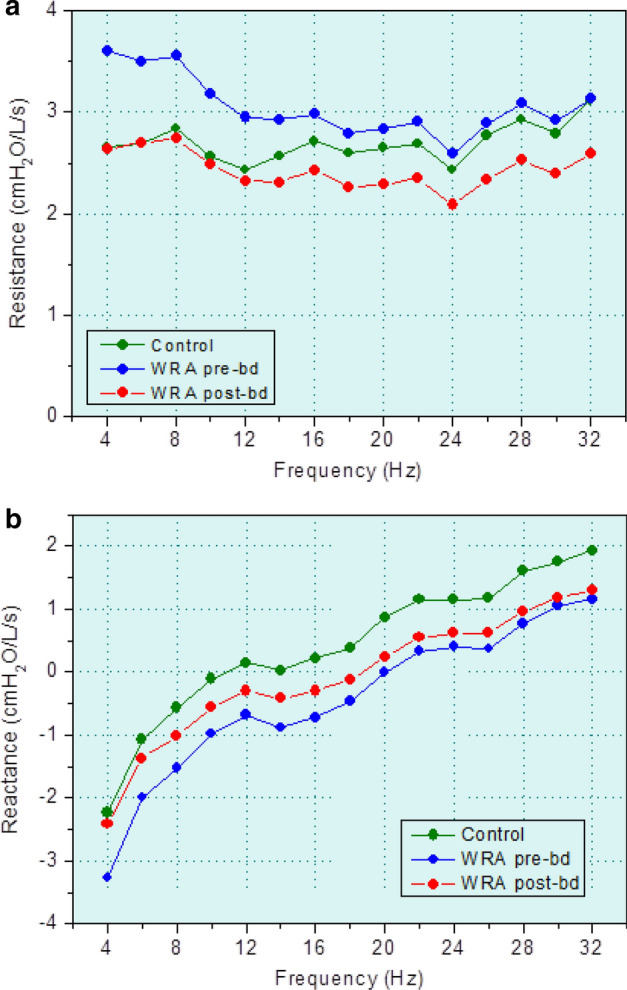
**a** Mean respiratory resistance and **b** reactance curves in controls and patients with work-related asthma (WRA) pre- and post-bronchodilator (BD) use

There is a consensus in the literature that FOT is in state of the art in the analysis of pulmonary function, contributing to increasing our knowledge about respiratory diseases, as well as in its diagnosis. However, only one study used this method to investigate WRA [3]. This study focused only on the evaluation of methacholine challenge and was also limited by the evaluation to just *R*_0_. Variables relating to respiratory reactance and respiratory modeling were not investigated. Supporting and adding new information to these previous results, Fig. 3a shows an increase in the respiratory obstruction in WRA. This increase was more discriminating in the 4–16 Hz range, which resulted in increased values of *R*_0_ and *R*_4_ (Table 3, Additional file 1: Figure S1), as well as highly significant reductions in ventilation homogeneity (*S* and *R*_4_ − *R*_20_). Considering the reactive changes (Fig. 3b), WRA introduced more negative values in reactance in comparison with the control group, which resulted in significant changes in all of the reactive parameters (Table 3). These results are consistent with preliminary results in a smaller group [32] and can be explained by the presence of bronchoconstriction and inflammatory processes in asthmatics. These abnormalities reduce the diameter of the internal airways introducing increased airways resistance and changes in the time constants in the ventilatory process of these patients.

One interesting finding was that *S*, *X*_m_, fr, Axt, and Axi presented fair values of AUC for clinical application (Table 7). These observations were in line with the usual interpretation of these parameters as being related to small airways disease [33–35] and the pattern of predominantly peripheral airway abnormalities in patients with mild obstruction, as may be characterized by the studied group.

Concerning the evaluation of the best methodology for calculating Ax, the correlations of Axi with spirometry (Table 5) and plethysmography (Table 6) were slightly higher than that obtained by Axt. Interestingly, the diagnostic accuracy of Axt in the identification of WRA respiratory abnormalities was higher than that observed in Axi (Table 7). It may be explained by the fact that, as can be seen in Fig. 3b, the reactance curve is not a perfect triangle. The approximation of the reactance area by a triangle amplifies the differences observed among the curves, improving the performance of Axt. Accordingly, Table 9 shows that the performance of Axt in the identification of the changes due to BD use was also higher than that obtained by Axi. Thus, although using a less accurate method to estimate area, Axt is more accurate than Axi in terms of clinical use. This counterintuitive finding may help elucidate the debate about the proper methodology for calculating Ax [8].

The bronchodilator use introduced a reduction in the resistance values and associated parameters (Fig. 3a, Table 3, and Additional file 1: Figure S1), as well as less negative values of respiratory reactance and parameters (Fig. 3b, Table 3). These results are in close agreement with the reduction in airway obstruction and the improvement in ventilation homogeneity usually observed after BD use in these patients [31].

**Fig. 4:**
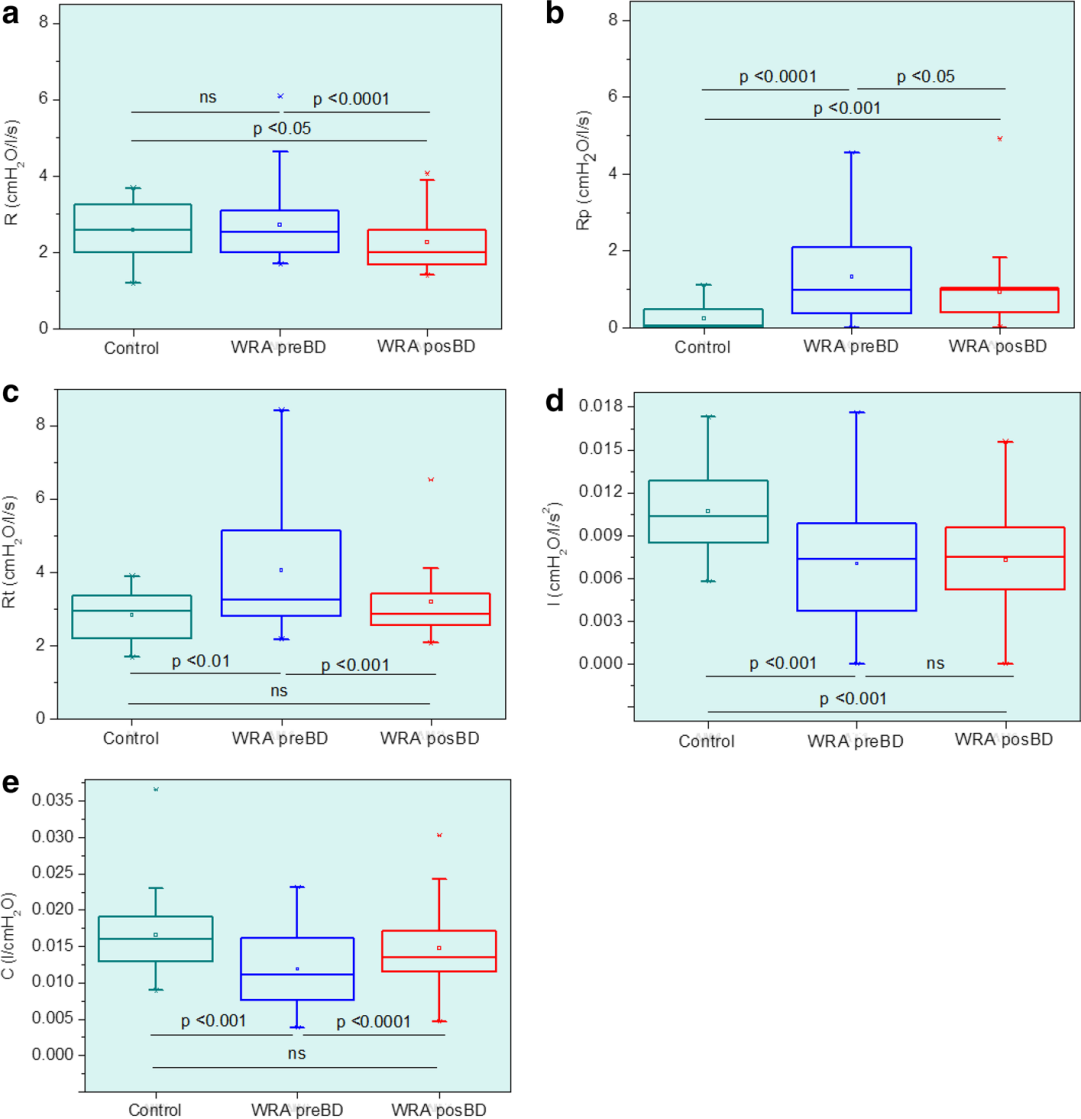
Influence of work-related asthma (WRA) and bronchodilator (BD) use on parameter values estimated from the eRIC model. Central airway resistance (*R*; **a**), peripheral resistance (*R*_p_; **b**), total resistance (*R*_t_; **c**), lung inertance (*I*; **d**), and alveolar compliance (*C*; **e**). The top and the bottom of the box plot represent the 25th- to 75th-percentile values, while the circle represents the mean value, and the bar across the box represents the 50th-percentile value. The whiskers outside the box represent the 10th- to 90th-percentile values

The effects of WRA and BD use in the parameters associated with the eRIC model are described in Fig. 4. WRA does not introduce alterations in *R*, which indicates small changes in the central airways of the studies patients. This result is consistent with the data obtained using spirometry and plethysmography (Table 2), which described the presence of a predominantly small or moderate obstruction in the studied sample. BD use resulted in a reduction in *R* (Fig. 4a). A possible explanation for this result may be the typical smooth muscle relaxation that occurs in these individuals. The resulting mean *R* values were smaller than that measured in controls. These findings are also in line with the predominantly small or moderate obstruction observed in the studied WRA population (Table 2).

Peripheral resistance increased in WRA (Fig. 4a), which could be attributed to inflammation and airway wall remodeling. These results are also likely to be related to airway smooth muscle shortening, which introduces peripheral constriction. It was interesting to note that, even after the BD use and a reduction in its value, the *R*_p_ of WRA patients remained higher than that observed in controls. This result is in contrast to the reduction of *R* to values smaller than those measured in controls (Fig. 4a), which is probably related to the inflammatory effect of the disease.

Figure 4c shows that WRA introduced increased values of *R*_t_. Considering that *R*_t_ = *R* + *R*_p_ [14] and that *R* was not increased in patients (Fig. 4a), we can speculate that this increase was associated with the increase observed in the peripheral resistance (Fig. 4b). This result reflects the fact that airway changes in asthma usually begin at the peripheral airways, and that the studied patients with WRA presented predominantly small or moderate obstruction (Table 2) so that they can be considered as in the early stages of the disease.

Respiratory inertance primarily describes the mass of gas that is moved during tidal breathing. It may be interpreted as an index related to pressure losses, as well, mostly due to the acceleration of the gas column in the central airways [8]. Respiratory inertance was reduced in WRA (Fig. 4d), which can be explained by the concepts of choke points [36] and apparent inertance [37]. Usually, inertance integrates the inertial characteristics of the whole respiratory system. As the respiratory obstruction advances, the oscillatory signal used by FOT to assess the impedance is prevented from passing through the choke points. It precludes FOT from considering the lung beyond the choke point so that the measured inertance reflects the airways proximal to the choke points. As a result, we observed a reduction in the apparent mass of the gas measured by the FOT, in the associated pressure necessary for the acceleration of the gas, and consequently, in the measured inertance. This process is similar to that observed in the apparent compliance and results in an apparent inertance. In line with this interpretation, direct associations were observed between inertance and spirometric indexes of peripheral airway obstruction (Table 5). Further additional supports to this hypothesis was provided by the inverse relationship obtained between inertance and the *R*_aw_ and the direct association observed with SGaw (Table 6). Bronchodilation does not cause a significant change in *I* (Fig. 4d). Similar to *S* (Additional file 1: Figure S1C), *R*_4_ − *R*_20_ (Additional file 1: Figure S1G), and *R*_p_ (Fig. 4b), *I* remained distinct from the results obtained in the control group after BD use. These results probably reflect the irreversible inflammatory effect of the disease.

WRA introduced a decrease in *C* (Fig. 4e). This finding is consistent with the work of Bhatawadekar et al. [38], which used a single compartment model fit to estimate Ers (1/Crs). These authors pointed out that Ers is associated with small airways, and potentially a very clinically useful measure in asthma. This parameter includes the lungs and bronchial wall compliances, the compliance of the chest wall/abdomen compartment, and the thoracic gas compression. Thus, this result may be related to airway remodeling and frequency dependence of dynamic compliance due to non-uniform ventilation. The deformation of the thoracic wall associated with lung hyperinflation also needs to be considered since it introduces an essential restrictive factor in the interaction between the lung and thoracic wall. In Fig. 4e, it is also apparent that the use of bronchodilator resulted in a significant increase in *C*, which became similar to that presented in regular patients. These results further support the idea of the reduction in airway obstruction and the improvement in ventilation homogeneity after BD use in these patients.

Considering the diagnostic use of eRIC parameters, only *R*_p_ reached an adequate value for clinical use (Table 8). This finding is in close agreement with the interpretation of this parameter as reflecting peripheral airway resistance, and the presence of peripheral changes in our studied patients, which shows mainly mild obstruction (Table 2).

**Fig. 5:**
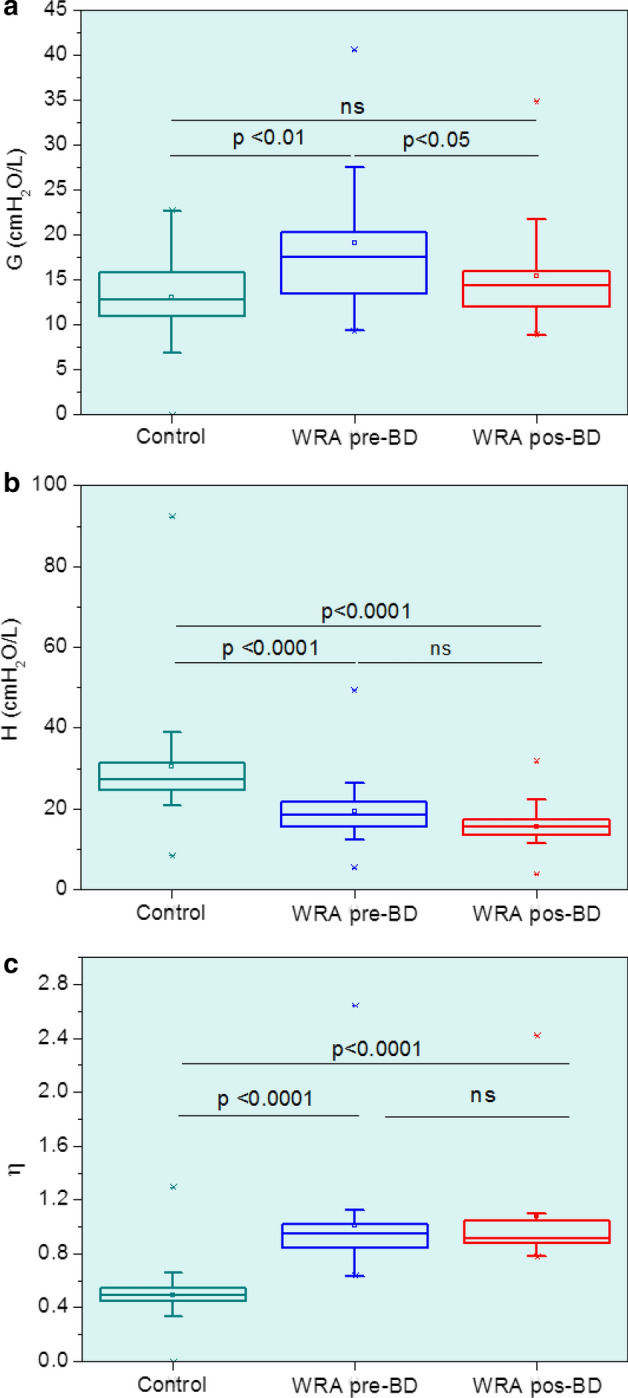
Impact of work-related asthma (WRA) and bronchodilator (BD) use on parameter values estimated from the fractional-order model. Respiratory damping (*G*; **a**), elastance (*H*; **b**), and hysteresivity (*η*; **c**). The top and the bottom of the box plot represent the 25th- to 75th-percentile values, while the circle represents the mean value, and the bar across the box represents the 50th-percentile value. The whiskers outside the box represent the 10th- to 90th-percentile values

Recently, the concept of FrOr modeling of the respiratory system has received significant interest in the research community [23, 28, 39–41]. Theoretically, these emerging models present an improved sensitivity to pathologic changes, due to an improved ability to capture the characteristics of respiratory mechanics. In reviewing the literature, however, no data were found on the question of FrOr modeling in patients with WRA. The current study found increased values of *G* in WRA, presenting a significant reduction after BD use (Fig. 5a). These findings broadly support the interpretation linking WRA with increased energy dissipation in the respiratory system [15], which may be explained by the increased airway obstruction and reduced respiratory compliance. This finding also supports evidence from clinical observations reporting increased respiratory work and dyspnea on small efforts in these patients. The reduction after BD use is also consistent with the reduction of dyspnea usually observed after BD use in these patients [1].

The current study observed values of *H* in controls (Fig. 5b) similar to that obtained previously [25, 26]. In contrast with the results previously reported in non-specific asthma [28], mild obstruction in patients with WRA introduced a highly significant reduction in *H*. This provides evidence that asthma resulting from occupational exposure results in more aggressive changes in terms of the elastic properties of the respiratory system than in the average of asthmatics. These findings may have essential implications in the development of objective methods for the differential diagnosis between WRA and non-work-related asthma.

It is also interesting to point out that *H* was also reduced in mild patients with other obstructive diseases, including chronic obstructive pulmonary disease [26], and silicosis [42], but not in asbestos-exposed workers with mild abnormalities [25]. This difference may be attributed to the restrictive nature of the asbestosis. It is a compelling finding since it provides another evidence that *H* may help in the differential diagnosis of work-related respiratory diseases.

Perhaps the most interesting finding in FrOr analysis was the increase in *η* values observed in patients with WRA (Fig. 5c). It is in close agreement with the involved physiology, reflecting chronic airway inflammation and remodeling, which predisposes the lung to a more heterogeneous pattern of peripheral airway constriction. A comparison between the present results and those of a preliminary study, including all asthma phenotypes [28], confirms the ability of this parameter to describe the presence of heterogeneous peripheral ventilation in the specific phenotype of WRA. Additional supports of this interpretation are provided by other studies performed recently in patients with sickle cell anemia [27], chronic obstructive pulmonary disease [22, 26], and asbestos-exposed workers [25]. The hysteresivity increases with the hysteresis area of the pressure–volume loop [43], which associates this parameter with the work of breathing [15, 21]. Correlation analysis was consistent with this interpretation, describing inverse associations with spirometric indexes of airway obstruction, and direct associations with *R*_aw_ (Tables 5 and 6, respectively). These findings indicate that *η* clearly describes the respiratory abnormalities in WRA, which are characterized by increased respiratory work [1].

Another interesting finding was the absence of changes in *η* values as a consequence of BD use (Fig. 5c). This result is in contrast with the reduction observed in *G* after BD use (Fig. 5a) and provided additional evidence of the association between *η* and peripheral abnormalities. Among FrOr parameters, *η* presented the highest correlations with spirometric and plethysmographic parameters of airway obstruction (Tables 5 and 6, respectively).

The range of measured values in asthmatics was reduced after BD use (Figs. 4 and 5). Patients with mild airway obstruction (61%) mainly compose the studied group of asthmatics. However, there are also 29% of patients with moderate and 10% with severe obstruction. This may explain the observed large range of measured values in asthmatics before BD use. After BD use, the airway obstruction tends to be reduced, and the respiratory system properties of the asthmatics tends to be closer to normal, reducing the range of measured values in these patients (Figs. 4 and 5).

It is now well established that fractional-order dynamic behavior may be linked to fractal structure, implying that properties of both function and structure are fundamentally interconnected [44]. It has been shown that recurrent fractal geometry may result in fractional-order terms [15]. In the particular case of the bronchial tree of normal subjects, a highly complex fractal structure is observed, in which the presence of self-similarity in its spatial structure is closely linked to a healthy lung function. In contrast, diseased lung presents asymmetry as a result of inhomogeneities due to the physiopathological process. The bronchial tree of a patient with WRA shows progressive loss of complexity in its spatial structure related to inflammation, airway remodeling, bronchoconstriction, edema, and fluid accumulation in the airways [31]. In line with these principles, previous studies from our laboratory demonstrate a consistent reduction in respiratory impedance complexity with increased airflow obstruction in a preliminary group, including all asthma phenotypes [45]. Further studies in similar groups of asthmatic subjects showed a significant increase in *η* and *G* with airway obstruction [28], indicating that these parameters are inversely related to respiratory complexity in these patients. It was hypothesized that the increase observed in *η* and *G* may be explained, at least partially, by the reduction in the complexity of the spatial structure of the airway tree of patients with asthma. Figure 5a, c provides further support to this hypothesis, extending this evidence to the specific case of WRA.

**Fig. 6:**
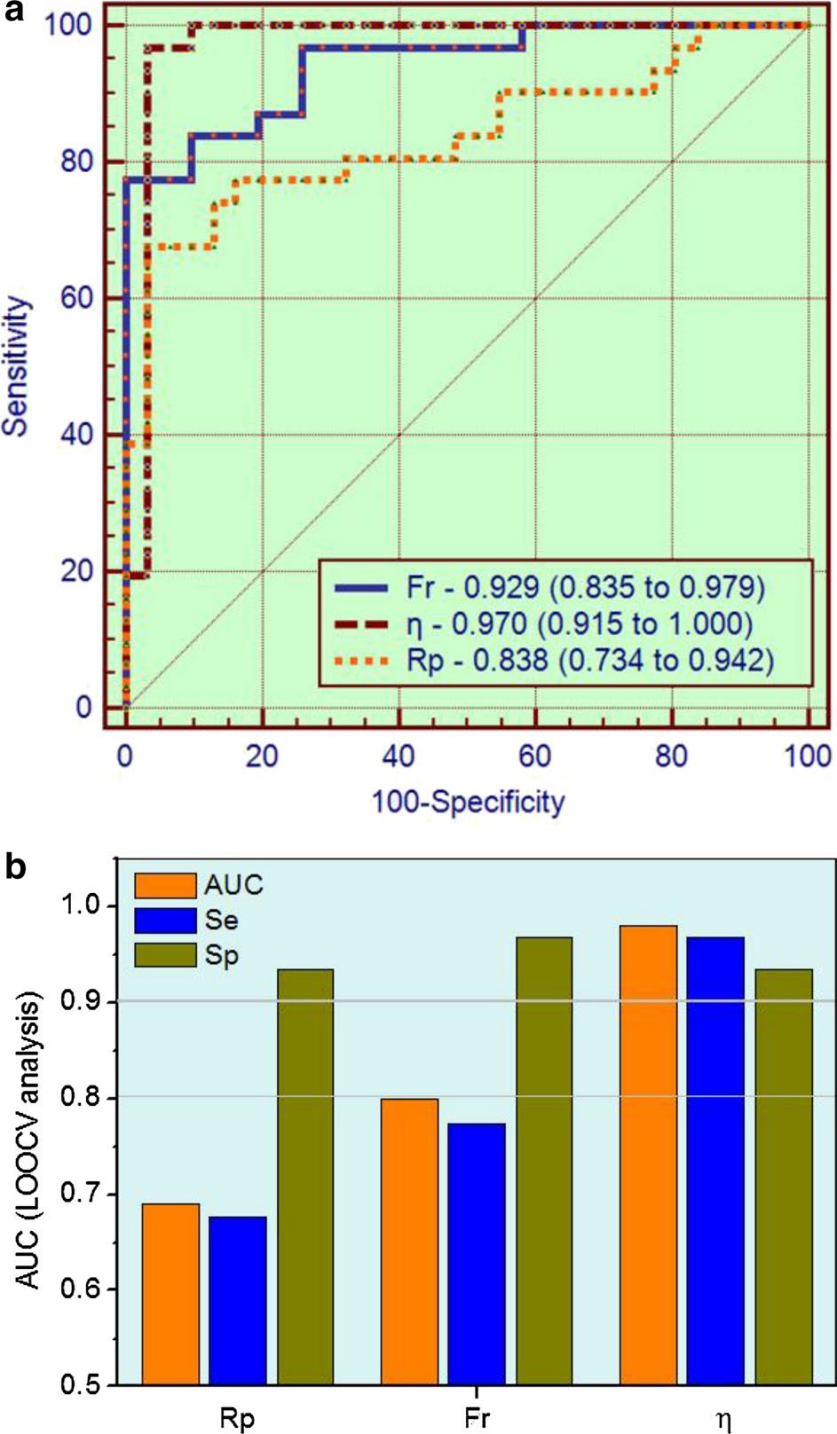
Receiver operator characteristic curves of the best parameters obtained from traditional FOT analysis, eRIC, and FrOr modeling to identify respiratory changes in WRA (**a**). Fr: resonance frequency; *R*_p_: peripheral resistance; *η*: hysteresivity coefficient, AUC: area under the receiver operating characteristic curve; Se: sensitivity, Sp: specificity. More restrictive leave-one-out cross-validation (LOOCV) analyses performed on the same parameters (**b**)

On the question of diagnostic use, this study found that *η*, obtained from FrOr modeling, reached a high diagnostic accuracy in identifying WRA abnormalities (Table 8). The comparison of the more accurate parameters obtained in traditional analysis, eRIC, and FrOr modeling showed that *η* was more accurate than *R*_p_ (Fig. 6). These results corroborate the propositions of previous authors, who suggested that FrOr models have the potential to improve respiratory clinical science and practice [10, 15, 16]. Also in line with this proposition and the observed results, it is apparent from the data in Table 4 that the FrOr model provided an improved description of the measured impedance. Following the present results, previous studies have demonstrated that FrOr models provide a more suitable fitting than integer-order models [26, 28]. It could be associated with the nature of the FrOr models, whose flexibility allows these models to adjust to fractional values of 20 dB/decade. Integer-order models, however, are only able to adjust to integer multiples of 20 dB/decade.

These results are in keeping with previous studies, in which the detailed analysis offered by FrOr modeling improved our knowledge about several biomedical areas, including the properties of the arterial wall in brain aneurysms [48], the description of the red blood cell membrane mechanics [49], and the blood flow in the cranial network [50]. Similar improvements were also observed in modeling the viscoelastic non-linear compressible properties of the lung [46], the blood ethanol concentration [47], and improving the chemotherapy used in cancer treatment [18].

The evidence presented in Table 8 and Fig. 6 supports the notion that FrOr models may be useful in clinical use. The increase in diagnostic accuracy obtained in the present study (Fig. 6) is in close agreement with improvements observed in the differentiation between malignant and benign breast lesions detected on X-ray screening mammography [48], cancer detection [49], screening for hemodialysis patients [50], differentiation of low- and high-grade pediatric brain tumors [51], and Parkinson’s disease severity assessment [52].

Regarding these aspects, an initially unexpected result was observed from data in Tables 9 and 10 and Fig. 2. In contrast to the results observed in the identification of changes in respiratory mechanics in WRA, there was no evidence that the parameters obtained from the FrOr model were more accurate than the traditional FOT parameters to identify the respiratory effects of BD use. When these results are analyzed more carefully, it can be observed that the highest accuracy observed among the traditional FOT parameters (Table 9) was obtained by *R*_4_, which is related to central airway obstruction. Among model parameters (Table 10), the most accurate was *R*. This eRIC model parameter also reflects mainly central airway resistance. Both results are in close agreement with the spirometric and plethysmographic changes observed after BD use in the present study (Table 2), which described changes associated mainly to central airways. These results are in line with the recent work of Bhatawadekar et al. [38] investigating the bronchodilator response in asthma. Thus, FrOr parameters were not the most adequate to diagnose these changes because these parameters are more related to peripheral airways, while the observed BD responses are involved with more central airways.

The findings in this study are subject to at least three limitations. First, the present work is limited to patients with WRA. This focus allowed us to investigate this specific phenotype, clarifying the use of FOT and respiratory modeling in this critical disease. However, many other types of asthma exhibit different features. Therefore, further studies are needed to assess these specific disorders.

Secondly, one could argue that the bronchodilator analysis was limited to evaluate the adequacy of the studied parameters to reflect the respiratory changes due to BD use. A comparative analysis of a group of BD responders and non-responders using spirometry as a reference and including the administration of placebo and BD medication in a large sample of patients could establish cut-off points for changes in parameters derived from FOT models. It has important practical application and should, therefore, be addressed in future studies.

Finally, the present study investigated a relatively small sample size. Although this limitation was minimized using the LOOCV method, it is still a limitation, and additional studies, including a more significant number of subjects, are necessary. The present analysis, however, significantly contributes to the essential debates in the literature concerning the proper methodology for calculating Ax [8], the use of FOT in occupational health [8], particularly in WRA [3], as well as introduced respiratory modeling in this disease.

## Conclusion

The present study provided clear evidence that patients with WRA show increased peripheral resistance, damping, and hysteresivity when compared with controls. These results provide new physiological insight into the effects of WRA on respiratory biomechanics. It was demonstrated that a combination of FOT and fractional-order modeling outperformed standard FOT, as well as integer-order modeling in the diagnosis of respiratory abnormalities in these patients, leading to high diagnostic accuracy. It was also shown that the use of bronchodilator in WRA resulted in increased dynamic compliance and reduced damping and peripheral resistance. FOT parameters may adequately identify these changes. Taken together, these results show the utility of the FOT associated with fractional-order modeling in the analysis of the respiratory abnormalities in patients with WRA.

## Materials and methods

The ethical investigation clearance was obtained from the Research Ethics Committee of the Pedro Ernesto University Hospital (protocol 456-CEP/HUPE). The study obeys the Declaration of Helsinki. Before data collection, the participants received an explanation of the project. Upon obtaining written informed consent from patients, the respiratory analysis was carried out.

### Study design, volunteers, and inclusion and exclusion criteria

This study utilized a cross-sectional design involving two groups of subjects: a group of patients with WRA and a group of controls. The asthmatics were diagnosed according to the GINA criteria [1]. These patients were not allowed to use the bronchodilator for at least 12 h before the test. They have not smoked for at least 2 h, nor have drunk coffee or alcohol for 6 h before the tests. The control group had no present or previous cardiorespiratory disease or medication, had no respiratory symptoms, and the findings in flow-volume spirometry were normal. For both groups, additional exclusion criteria were tuberculosis, inability to perform the tests, chemotherapeutic or radiotherapeutic treatment, and respiratory infections in the last 30 days.

The WRA group measurements were analyzed for two moments: pre-bronchodilator (WRA Pre-BD) and post-bronchodilator (WRA Post-BD). The use of bronchodilator medication (400 μg salbutamol sulfate spray) was performed immediately after the first set of pulmonary function tests. The second group of tests was performed 15 min later.

### Forced oscillation

FOT was evaluated using small amplitude pressure oscillations (≤ 2 cmH_2_O) generated by a speaker applied during spontaneous breathing at the entrance of the airway through the oral cavity. Three tests were conducted, each 16 s long, with the mean score being adopted as the final result. The test was considered acceptable if the volunteers presented stable tidal volumes and rate and free of pauses. Common artifacts, such as swallows, coughs, and leaks, were identified by the evaluation of flow and pressure signals. The acquisition was repeated until three stable and free of artifact measurements were obtained. The forced pseudo-random noise used in this study was composed of a frequency range between 4 and 32 Hz. To reduce the influence of the spontaneous breathing signal in the lowest frequency range, the minimum coherence function (CF) used for the acceptance of results was 0.9. The exams are repeated until all analyzed frequencies have this minimum CF value. To exclude outlying values, the coefficient of variability at the lowest oscillation frequency (4 Hz) of the three used tests was ≤ 10%. The analyses were performed using an *OSCILAB 2.0* impedance analyzer developed at our laboratory [53].

The real part of impedance was submitted to linear regression analysis in the 4–16 Hz range, which yielded respiratory resistance extrapolated at 0 Hz (*R*_0_), and frequency dependence of *R*_rs_ was expressed as the slope (*S*) of the linear relationship between the resistive impedance and frequency. The mean resistance (*R*_m_) in this frequency range was also evaluated. *R*_0_ is related to the low-frequency range. Newtonian resistance of the respiratory system, including the airways and resistance of tissue originating from the lung and chest wall, along with the effect of gas redistribution (pendelluft) [54]. *S* describes the resistance change with frequency and is related to respiratory system non-homogeneities [55]. These non-homogeneities are associated with increased peripheral resistance. The wide range of frequencies used in FOT allows for independent assessment of both proximal and peripheral airways. Lower frequencies (e.g., 4 Hz) penetrate deeply in the lung structure and are related to the sum of the proximal and peripheral airways. As the frequency rises, the measurement signal penetrates less and less into the lung structure, so that at higher frequencies (e.g., 20 Hz) is related only to proximal airways. Consequently, the resistance change with frequency has been used as a surrogate marker to describe peripheral resistance. *R*_m_ describes the mid-frequency range resistance, which is sensitive to the airway caliber, reflecting resistance in the central airways [56]. Other usual indexes of respiratory resistance were also studied; the resistances in 4 Hz (*R*_4_), 12 Hz (*R*_12_), and 20 Hz (*R*_20_), representing the low-, mid-, and high-frequency spectra, respectively, and the frequency dependence of resistance, which was represented as the difference between *R*_4_ and *R*_20_ (*R*_4_ − *R*_20_).

The results associated with the imaginary part of the impedance were interpreted using five parameters: mean reactance (*X*_m_), resonance frequency (fr), respiratory system dynamic compliance (*C*_dyn_), the impedance module, and the area under the reactance curve. Mean reactance is generally related to respiratory system inhomogeneity, and in this study, it was calculated using the 4 to 32 Hz frequency range. The frequency at which *X*_rs_ becomes zero is known as the resonance frequency [57]. *C*_dyn_ is related to the total compliance of the respiratory system, comprising pulmonary compliance, chest wall compliance, and airway compliance. This parameter is also related to the homogeneity of the respiratory system [56]. *C*_dyn_ was calculated based on the reactance at 4 Hz (*C*_dyn_ = 1/2*π*f*X*_4_). The 4 Hz impedance module (*Z*_4_) was also studied, which reflects the total mechanical load of the respiratory system [58].

The area under the reactance curve from the lowest frequency to fr (Ax) is also reported. There is a debate about the proper methodology for calculating Ax that has gained recent prominence [8], which includes the frequency resolution and numerical integration method as essential points to be clarified. To help address these research gaps, we evaluated Ax using two different methods. First, we used the approximation of the area by a triangle (AXt) defined by the lowest frequency studied (4 Hz), the corresponding reactance value (*X*_4_), and the resonance frequency. The second method uses the integral based on the trapezoidal rule to obtain a more accurate area estimate (Axi).

### Spirometry and plethysmography

After FOT, spirometry was performed and interpreted according to the recommendations of the American Thoracic Society/European Respiratory Society [59]. Plethysmographic exams were conducted using the reference values described by Ref. [60] and a constant volume and variable pressure plethysmograph (HD CPL nSpire Health Ltd., Hertford, UK). The studied parameters were the airway resistance (*R*_aw_), the total lung capacity (TLC), functional residual capacity (FRC) and residual volume (RV), as well as their relationships (RV/TLC and FRC/TLC).

### Integer-order modeling

The extended resistance–inertance–compliance (eRIC) compartmental model was used to interpret the changes in the respiratory system impedance due to the presence of WRA (Fig. 1). The cited model was introduced as an improved version of the basic RIC model [10].

This model offers a detailed description of the respiratory system properties, using *R* to describe the central airway resistance, while the peripheral resistance (*R*_p_) is associated with the small airways. *R*_p_ allows for the frequency dependence of resistance values typically observed in patients, which is beyond the basic RIC capability. Reactive properties are described in this model by the respiratory inertance (*I*) and compliance (*C*) [14].

### Fractional-order modeling

**Fig. 7:**
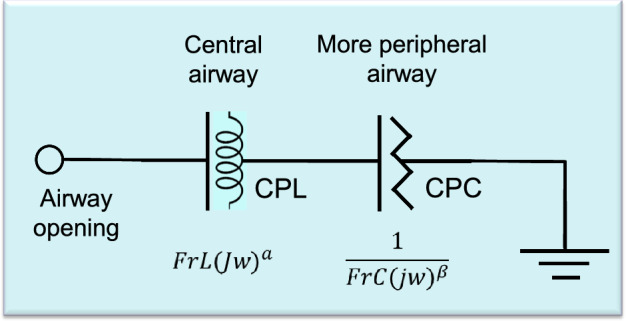
Fractional-order model based on a series association of a constant phase inertance (CPL) and constant phase compliance (CPC). These elements are composed of a frequency-dependent fractional inertia (FrL) and a frequency-dependent fractional compliance (FrC) elements, both related to their associated fractional exponents *α* and *β*

A recently described [23] fractional model of the respiratory impedance (*Z*_FrOr_) was used, according to Eq. (1):

1 $$ Z_{\text{FrOr}} \left( {j\omega } \right) = {\text{FrL}}\left( {j\omega } \right)^{\alpha } + \frac{1}{{{\text{FrC}}\left( {j\omega } \right)^{\beta } }}, $$

where FrL represents a frequency-dependent fractional-order inertance, associated with a fractional inertance coefficient (0 ≤ *α* ≤ 1), while FrC represents the constant-phase fractional-order compliance, which in turn is associated with a fractional compliance coefficient (0 ≤ *β* ≤ 1). Figure 7 depicts this model.

Figure 7 shows that the coupling of airway structures with dissipative energy (resistive), as well as storage energy properties (inertial), is described by FrL. The associated *α* coefficient modulates the influence of FrL in the frequency domain. As *α* approaches to zero, the influence of FrL in the airway resistance increases, with a concomitant reduction of its influence in airway inertance. Considering the impedance curves, it describes increased resistance values with frequency and more negative values of reactance in higher frequencies.

As described in Fig. 7, more peripheral structures of the respiratory system were described as the constant-phase fractional-order compliance (FrC). This parameter reflects structures that elastically store energy, and that is also simultaneously coupled to those that dissipate energy. The influence of FrC in resistance and reactance is modulated by *β*, where lower values are related to increased resistance and reduced compliance. In impedance traces, it results in increased resistance values, as well as more negative values of reactance in the low-frequency range.

The cited parameters will change according to the physiological relations of the respiratory system, involving morphology and geometry [15]. The damping factor (*G*) is usual in the interpretation of these physiological relations, representing the energy dissipation in the respiratory system [15]. This parameter is defined as follows:

2 $$ G = \frac{1}{C}\cos \left( {\frac{\pi }{2}\beta } \right). $$

The elastance (*H*) is a measure of potential elastic energy accumulation, according to Eq. (3):

3 $$ H = \frac{1}{C}\sin \left( {\frac{\pi }{2}\beta } \right). $$

Another widely used parameter is the hysteresivity coefficient (*η*), which is proportional to the heterogeneity of ventilation in the lung [15] and defined as

4 $$ \eta = \frac{G}{H}. $$

The fitting of the selected parameters was implemented using the Levenberg–Marquardt algorithm, determining the set of parameters of the model that best represents the input dataset in terms of least squares. The measured FOT data in the frequency range between 4 and 32 Hz was used as input dataset. The software for curve fitting was developed in the LABVIEW™ 12.0 (National Instruments, Austin, TX) environment, providing as a measure of the goodness of fit of the model the total error value (MSEt). This parameter was calculated as the square root of the sum of the real (MSEr) and imaginary (MSEx) impedance estimation errors. Following the procedure used by Oostveen et al. [61], a further error analysis was performed using the mean relative distance from the model and measured resistance and reactance values (*R*_d_).

### Statistical analysis

A commercial software (Origin^®^ 8.0, Microcal Software Inc., Northampton, Massachusetts, United States) was used to assess normality (Shapiro–Wilk test) and to perform statistical tests (*t* test or Mann–Whitney). The results are presented as the mean ± SD, and statistical significance was considered when *p* ≤ 0.05. The association of model parameters and pulmonary function was investigated using the correlation analysis. Pearson or Spearman correlation was used depending on whether data distribution is normal or not. The following categorization of the strength of these associations was used [62]:

- Small or no correlation (− 0.25 to 0.25);
- Reasonable correlation: (0.25 to 0.50, or − 0.25 to − 0.50);
- Moderate to good correlation: (0.50 to 0.75, or − 0.50 to − 0.75);
- Very good to excellent correlation: (0.75 to 1, or − 0.75 to − 1).

It was used a correction in the significance level to minimize the chances of making a Type I error (modified Bonferroni) due to the computation of several correlations [63]. This correction was performed dividing the *p*-value by an estimate of the effective number of independent correlations used. Usually, four independent variables are observed in traditional pulmonary function exams, while two independent variables are associated with the resistive and reactive properties of the FOT. Thus, a corrected significance level of 0.0063 (0.05/8), associated with eight independent correlations, was used.

Receiver operating characteristic (ROC) curves were used to evaluate the clinical potential of the FOT indexes. The values of sensitivity, specificity, and area under the curve (AUC) were obtained based on the optimal cut-off point, as determined by the ROC curve analysis. MedCalc 12 (MedCalc Software, Mariakerke, Belgium) was used in these analyses, which followed the STARD [64] requirements for studies of diagnostic accuracy. Leave-one-out cross-validation (LOOCV) was employed to minimize the statistical problem of finite patients. These evaluations were conducted as described in Ref. [65].

The minimum value of the AUC considered adequate for the identification of the changes due to WRA was 0.8 [66, 67]. Following previous studies concerning the use of bronchodilators in asthmatics [68, 69], 0.7 was considered to be a reasonable cut-off value for the diagnosis of respiratory changes due to BD use.

The sample size was calculated using MedCalc 12.3 (MedCalc Software, Mariakerke, Belgium). The used criterium was the comparison of the area under a ROC curve with a null hypothesis value [70]. Here, the objective was to show that adequate diagnostic accuracy (AUC = 0.8) [66, 67] was significantly different from the null hypothesis (AUC = 0.5), which indicates no clinical diagnostic value. The initial results obtained in a pilot study, including 17 controls and 12 patients [32], were used. Considering adequate type I and type II errors of 0.10, this analysis resulted in a minimum of 29 volunteers per group.

## Supplementary information

**Additional file 1: Figure S1.** Comparative analysis of the traditional FOT resistive parameters obtained in controls and patients with work-related asthma (WRA) pre and post bronchodilator use: resistance in 0 Hz (*R*_0_; A), mean resistance (*R*_m_; B), slope of the resistance values (*S*; C), resistance in 4 Hz (*R*_4_; D), resistance in 12 Hz (*R*_12_; E), resistance in 20 Hz (*R*_20_; F) and difference in the resistance 4 Hz and 20 Hz (*R*_4_ − *R*_20_; G).

**Additional file 2: Figure S2.** Comparative analysis of the traditional FOT reactive parameters obtained in controls and patients with work-related asthma (WRA) pre and post bronchodilator (BD) use: mean reactance (*X*_m_; A), resonant frequency (Fr; B), dynamic compliance (*C*_dyn_; C), reactance area approximated by a triangle (Axt: D), by an integral (Axi; E), impedance module in 4 Hz (*Z*_4_; F).

## Data Availability

The datasets used or analyzed during the current study are available from the corresponding author on reasonable request.

## Abbreviations

*α*: Fractional inertance coefficient
*β*: Fractional compliance coefficient
*η*: Hysteresivity coefficient
AUC: Area under the receiver operating characteristic curve
Axi: Area under the reactance curve using the integral based on the trapezoidal rule
Axt: Area under the reactance curve using the approximation by a triangle
BD: Bronchodilator
*C*: Compliance
*C*_dyn_: Dynamic compliance
CF: Coherence function
EOA: Exacerbated occupational asthma
eRIC: Extended resistance–inertance–compliance model
FEV_1_: Forced expiratory volume in the first second
FOT: Forced oscillation technique
fr: Resonant frequency
FrC: Fractional-order compliance
FRC: Functional residual capacity
FrOr: Fractional-order
FrL: Fractional-order inertance
FVC: Forced vital capacity
*G*: Damping factor
*H*: Elastance
*I*: Respiratory inertance
LOOCV: Leave-one-out cross-validation
MSEr: Real mean square error value
MSEt: Total mean square error value
MSEx: Imaginary mean square error value
OA: Occupational asthma
*R*: Central airway resistance
*R*_p_: Peripheral resistance
*R*_0_: Intercept resistance
*R*_4_: Respiratory resistance in 4 Hz
*R*_12_: Respiratory resistance in 12 Hz
*R*_20_: Respiratory resistance in 20 Hz
*R*_4_ − *R*_20_: Difference between the respiratory resistance in 4 Hz and 20 Hz
*R*_aw_: Airway resistance
*R*_d_: Mean relative distance from the model and measured resistance and reactance
*R*_m_: Mean resistance in the 4–16 Hz range
ROC: Receiver operating characteristic curve
*R*_rs_: Respiratory resistance
RV: Residual volume
*S*: Angular coefficient of resistance in the 4–16 Hz range
Se: Sensitivity
Sp: Specificity
TLC: Total lung capacity
WRA: Work-related asthma
*X*_4_: Respiratory reactance at 4 Hz
*X*_m_: Mean reactance evaluated considering the 4 to 32 Hz frequency range
*X*_rs_: Respiratory reactance
*Z*_4_: Impedance module in 4 Hz
*Z*_FrOr_: Fractional-order respiratory impedance
