# Supplementary material for: Combined forced oscillation and fractional-order modeling in patients with work-related asthma: a case–control study analyzing respiratory biomechanics and diagnostic accuracy
Source: Biomed Eng Online. 2020 Dec 9;19:93. doi: 10.1186/s12938-020-00836-6 (PMC7724713; doi:10.1186/s12938-020-00836-6)
Supplement: Supplementary file 1 — Additional file 1: Figure S1. Comparative analysis of the traditional FOT resistive parameters obtained in controls and patients with work-related asthma (WRA) pre and post bronchodilator use: resistance in 0 Hz (R0; A), mean resistance (Rm; B), slope of the resistance values (S; C), resistance in 4 Hz (R4; D), resistance in 12 Hz (R12; E), resistance in 20 Hz (R20; F) and difference in the resistance 4 Hz and 20 Hz (R4 − R20; G). [file 12938_2020_836_MOESM1_ESM.pdf]

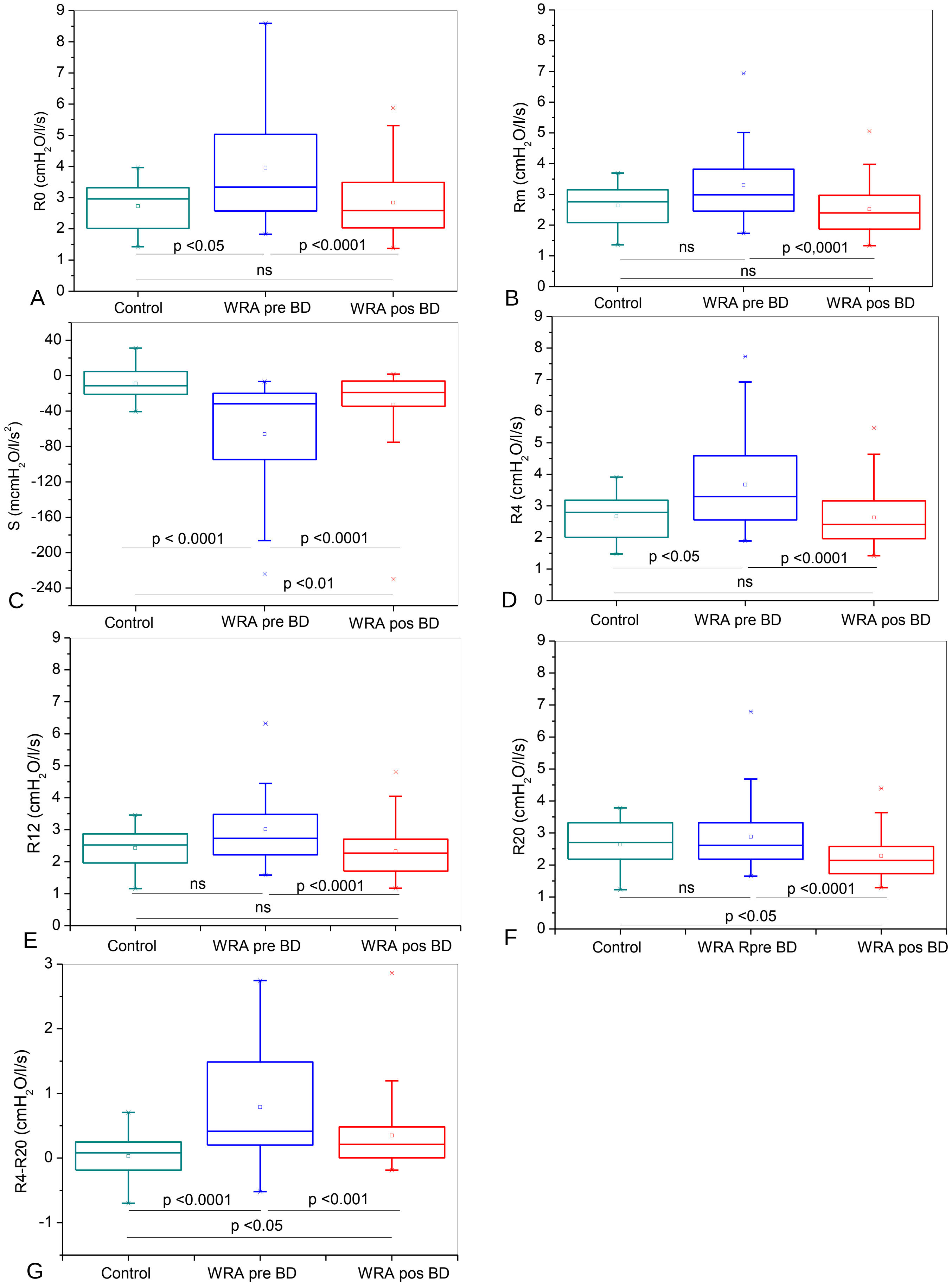

Figure S1 – Comparative analysis of the traditional FOT resistive parameters obtained in controls and patients with work-related asthma (WRA) pre and post bronchodilator use: Resistance in 0 Hz (R0; Figure A), mean resistance (Rm; Figure B), slope of the resistance values (S; Figure C), resistance in 4 Hz (R4; Figure D), resistance in 12 Hz (R12; Figure E), resistance in 20 Hz (R20; Figure F) and difference in the resistance 4 Hz and 20 Hz (R4-R20; Figure G).
