# Supplementary material for: Combined forced oscillation and fractional-order modeling in patients with work-related asthma: a case–control study analyzing respiratory biomechanics and diagnostic accuracy
Source: Biomed Eng Online. 2020 Dec 9;19:93. doi: 10.1186/s12938-020-00836-6 (PMC7724713; doi:10.1186/s12938-020-00836-6)
Supplement: Supplementary file 2 — Additional file 2: Figure S2. Comparative analysis of the traditional FOT reactive parameters obtained in controls and patients with work-related asthma (WRA) pre and post bronchodilator (BD) use: mean reactance (Xm; A), resonant frequency (Fr; B), dynamic compliance (Cdyn; C), reactance area approximated by a triangle (Axt: D), by an integral (Axi; E), impedance module in 4 Hz (Z4; F). [file 12938_2020_836_MOESM2_ESM.pdf]

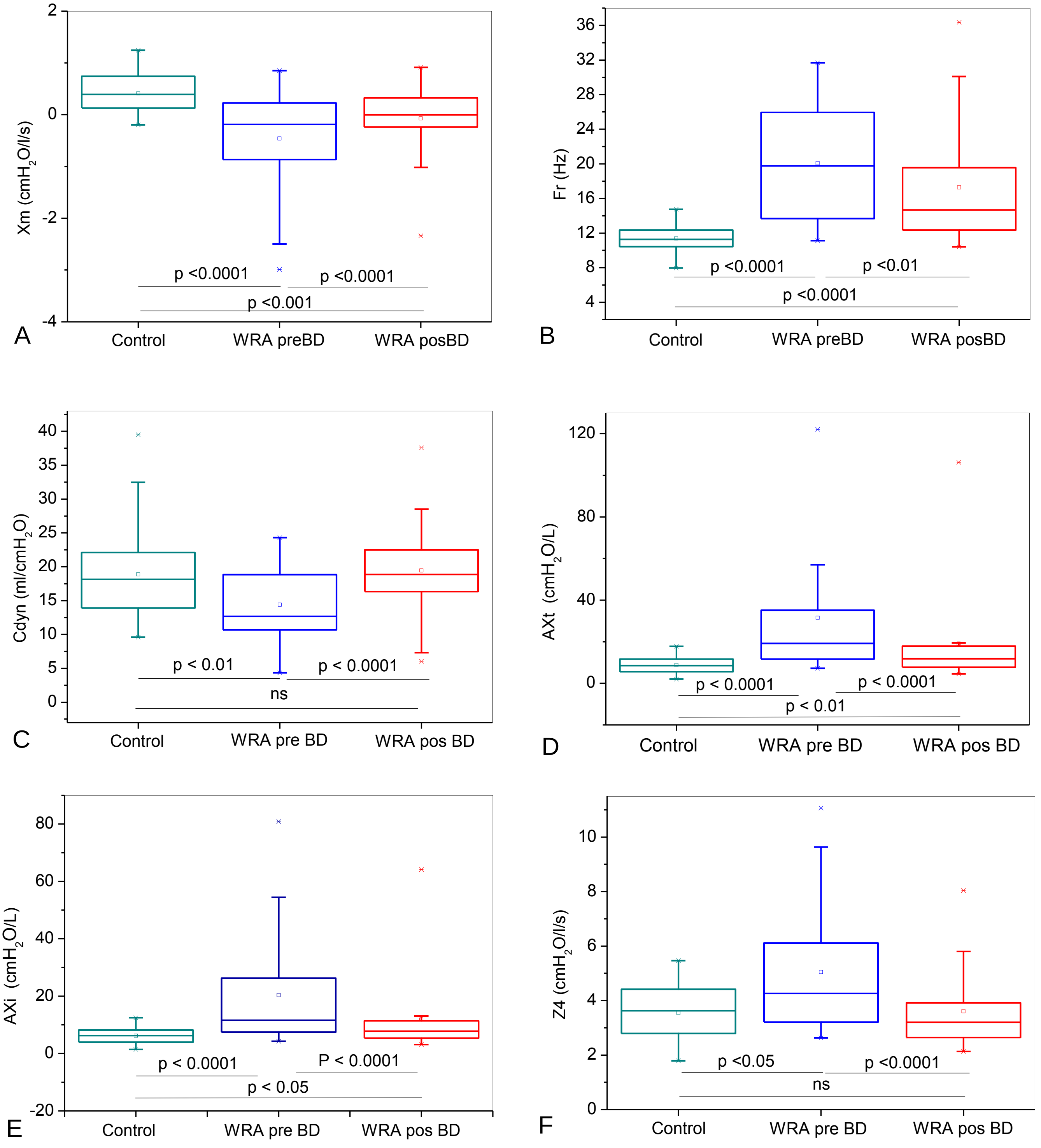

Figure S2 – Comparative analysis of the traditional FOT reactive parameters obtained in controls and patients with work-related asthma (WRA) pre and post bronchodilator (BD) use: Mean reactance (Xm; Figure A), resonant frequency (Fr; Figure B), dynamic compliance (Cdyn; Figure C), reactance area approximated by a triangle (Axt: Figure D), by an integral (Axi; Figure E), impedance module in 4 Hz (Z4; Figure F).
